# Requirements for Membrane Attack Complex Formation and Anaphylatoxins Binding to Collagen-Activated Platelets

**DOI:** 10.1371/journal.pone.0018812

**Published:** 2011-04-15

**Authors:** Catherine Martel, Sylvie Cointe, Pascal Maurice, Saïd Matar, Marta Ghitescu, Pierre Théroux, Arnaud Bonnefoy

**Affiliations:** 1 Department of Medicine, Montreal Heart Institute and University of Montreal, Montreal, Quebec, Canada; 2 INSERM U743, University of Montreal, Montreal, Quebec, Canada; French National Centre for Scientific Research, France

## Abstract

**Background:**

The activation of complement during platelet activation is incompletely understood. *Objectives*: We sought to explore the formation of C5b-9 and anaphylatoxins binding to collagen-activated platelets.

**Methods:**

C5b-9, anaphylatoxins C3a, C4a and C5a, and anaphylatoxin receptors C3aR1 and C5aR were measured by flow cytometry and/or confocal microscopy. Platelet microparticles were quantified by flow cytometry, and their C5b-9 content was determined by western blot analyses. In all experiments, sodium citrate was used for blood anticoagulation.

**Results:**

C5b-9 rapidly formed on the platelet surface following activation with collagen, TRAP, ADP or A23187, but was surprisingly restricted to a subset of platelets (1 to 15%) independently of P-selectin or phosphatidylserine exposure. Following collagen activation, C5b-9-positive platelets in thrombi were found associated with collagen fibres. C5b-9 formation was obliterated by Mg^2+^-EGTA and significantly reduced by the thrombin inhibitor hirudin (−37%, p<0.05), but was unaffected by chondroitinase, compstatin, SCH79797 (PAR-1 inhibitor), or in the PRP of a MBL-deficient donor. Compstatin and Mg^2+^-EGTA, but not hirudin, SCH79797 or chondroitinase, inhibited the formation of collagen-induced microparticles (−71% and −44%, respectively, p<0.04). These microparticles contained greater amounts of C5b-9 compared with the other agonists. Platelet activation by collagen or convulxin resulted in the strong binding of anaphylatoxins and the exposure of receptors C3aR1 and C5aR (CD88) on their surface.

**Conclusions:**

C5b-9 formation on collagen-activated platelets is i) partially controlled by thrombin, ii) restricted to a subset of platelets, and iii) can occur without P-selectin expression or phosphatidylserine exposure. Activated platelets bind anaphylatoxins on their surface and express C3a and C5a receptors, which may contribute to the localization of inflammatory processes during thrombosis.

## Introduction

There is a growing body of evidence supporting a bridging role of the complement system (CS) between inflammation and thrombosis through the activation of platelets. Hence, thrombin-mediated platelet aggregation and secretion are enhanced by complement components [1], most of them (C5, C6, C7, C8, C9 and, to a lesser extent, C3) being stored in platelets and secreted following activation [2], [3]. The insertion of C5b-9 in the platelet membrane induces reversible membrane depolarisation [4], phosphatidylserine exposure [5], microparticle formation and catalysis of prothrombinase activation in a calcium- and protein kinase-dependent manner [6], [7]. Theses mechanisms contribute to the activation and the propagation of the coagulation cascade.

On the other hand, several groups have recently demonstrated that platelet activation induces complement activation via different mechanisms [8]. Platelet P-selectin can bind C3b thereby initiating the activation of the complement alternative pathway (AP) [9], while the classical pathway (CP) is activated on the platelet surface *via* a C1q and gC1qR/p33-dependent activation of C4 [10]. Finally, chondroitin sulfate secreted by thrombin-activated platelet was found to trigger the fluid phase activation of the classical pathway in a C1q-dependent manner [11].

Complement activation may have fundamental implications in immuno-inflammation-induced damage during acute coronary syndromes and microangiopathies, such as in paroxysmal nocturnal hemoglobinuria and hemolytic uremic syndrome. However, the kinetics and extent of C5b-9 formation on activated platelets are largely uncharacterized, and the mechanisms of complement activation by activated platelet remain incompletely understood. Using a panel of platelet and complement inhibitors, the present study aimed at characterizing the requirements for membrane attack complex formation and anaphylatoxins binding to collagen-activated platelets.

## Methods

### Blood sampling

This study followed the guidelines of and was approved by the ethics committee of the Montreal Heart Institute, and written informed consent was obtained from all participants included in the study. Twenty mL of blood were drawn from the antecubital vein of healthy volunteers free from any medication known to interfere with platelet function and complement activation. The first 2 mL were discarded and the remaining 18 mL were transferred in plastic tubes containing 0.105 M sodium citrate. As determined by functional solid phase assays of C5b-9 formation (Wielisa kit COMPL 300 Wieslab, Lund, Sweeden) [12], sodium citrate was selected as the blood anticoagulant over heparin, ethylenediaminetetraacetic acid (EDTA) and D-phenylalanyl-L-prolyl-L-arginine chloromethyl ketone (PPACK) because of its lower impact on the three complement activation pathways (see Supporting Information S1).

### Platelet aggregation

Citrated blood was centrifuged at 130 g for 15 min at room temperature (RT). Platelet-rich plasma (PRP) was harvested, and the remaining blood was centrifuged at 1800 g for 15 min at RT to obtain platelet-poor plasma (PPP). Aggregations of PRP adjusted to 250.10^3^ platelets/µL were performed for 30 seconds, 5 and 10 min at 37°C with a Chrono-log aggregometer 570 (Chrono-log Corporation, Havertown, PA, USA) following activation with either 5 µM adenosine diphosphate (ADP), 10 µM Thrombin Receptor Agonist Peptide (TRAP) (Sigma Chemical Co, St-Louis, MO.), 2.5 µg/mL collagen (native, acid-insoluble fibrillar equine type 1 collagen, Chronolog, Havertown, USA) or 50 µM calcium ionophore A23187. Any further complement activation was then blocked by adding 50 µg/mL of the broad serine protease inhibitor Nafamostat Mesilate (FUT-175, BD Pharmingen, Mississauga, ON, Canada). Non-activated PRP was used as a negative control for both platelet and complement activation.

In another series of experiments, PRPs were activated for 10 min under stirring conditions with 2.5 µg/mL fibrillar collagen in the presence of either FUT-175, 10 mM ethylene glycol tetraacetic acid plus 2.5 mM Mg^2+^ (Mg^2+^-EGTA), 5 U chondroitinase ABC (Sigma Aldrich, Oakville, ON, Canada), 10 U/mL hirudin (Calbiochem, CA, USA), 3 µM of the protease activated receptor-1 (PAR-1) inhibitor SCH79797, or 100 µM of the C3 inhibitor compstatin (both from Tocris Biosciences, Ellisville, MO, USA). In one experiment, the PRP from a mannan-binding lectin (MBL)-deficient donor was used (MBL value in serum below 100 ng/mL) to obtain some insight on the involvement of the lectin pathway (LP).

### C5b-9 formation on platelet aggregates

Aggregated PRPs were centrifuged at 1800 g for 15 min, and the supernatants were harvested and frozen immediately at −70°C for further analyses. The platelets were resuspended in a phosphate buffer saline containing 1% bovine serum albumin (BSA) and 10 mM EDTA (PBS-EDTA).

The formation of C5b-9 on aggregated platelets was assessed by flow cytometry (EPICS XL, Beckman Coulter, Florida, USA) and confocal microscopy (Zeiss Observer Z1 equipped with a Yokogawa CSU-X1 confocal head and a Cascade QuantEM 512SC Camera, Intelligent Imaging Innovation Denver, CO, USA). A mouse anti-human antibody (DakoCytomation, Denmark) recognizing a neoepitope on C9 that only appears following the incorporation of C9 into C5b-9 was used (clone AE11 which does not cross react with goat C9). Platelet surface was saturated with 1% heat-inactivated goat serum in PBS-EDTA and incubated for 30 min at RT with the anti-C5b-9 antibody. After centrifuging at 500 g for 10 min, an Alexa Fluor® 488-goat anti-mouse IgG (Invitrogen, Burlington, ON, Canada) was added to the aggregates and incubated for 30 min at RT. After washing twice with 140 mM NaCl, 10 mM HEPES, 2.5 mM CaCl_2_, pH 7.4, the platelets were stained with an Alexa Fluor 647-mouse anti-human P-selectin antibody and Pacific Blue®-Annexin V (BioLegend, San Diego, CA, USA) for flow cytometry or confocal microscopy analyses. Confocal images were processed for iterative deconvolution before analysis. The C5b-9 signal measured by flow cytometry was adjusted by subtracting the fluorescence of a non-immune IgG. To measure the density of the C5b-9 fluorescent signal in both platelets and aggregates, the ratio between the adjusted mean fluorescence intensity (MFI) and the mean forward scatter (FSC) was calculated, as described elsewhere [13].

### Anaphylatoxins measurement

In a first series of experiments, the plasmas from activated PRPs were thawed at 4°C overnight and centrifuged at 8000 g for 5 min. C3a, C4a and C5a were measured by flow cytometry with the Cytometric Bead Array immunoassay (CBA, Becton Dickinson^TM^, San Diego, CA, USA). Briefly, the samples were incubated for 2 hrs with specific anti-anaphylatoxin antibody-coupled beads, washed, incubated for 1 h with a mixture of Phycoerythrin-conjugated C3a, C4a and C5a antibodies, and immediately measured. The sensitivity of the assay was 4, 10.5 and 1.2 ρg/mL for C3a, C4a and C5a, respectively. In a second series of experiments, collagen-induced platelet aggregates formed in 250 µL of PRP were washed, resuspended in 35 µL PBS-EDTA and incubated for 30 min at RT with 35 µL of the above anti-anaphylatoxin antibody cocktail. Platelet-bound anaphylatoxins were detected by fluorescence microscopy measuring the mean fluorescence density of the thrombi. In a third series of experiments, collagen-induced platelet aggregates formed in 250 µL of PRP were washed, resuspended in 35 µL PBS-EDTA, and incubated for 30 min at RT with 5 µl of either the anti-C3a, anti-C4a or anti-C5a antibody-coupled beads (previously washed and concentrated 30 times in PBS) from the CBA assay. Platelet-bead interaction was then measured by contrast phase microscopy.

### Detection of anaphylatoxin receptors C3aR1 and C5aR (CD88) on collagen-activated platelets

PRP acidified with acid citrate dextrose (ACD) formula A 1/10 (v/v) (Baxter) was centrifuged at 800 g for 15 min at RT. Platelets were resuspended in Tyrode's buffer (137 mM NaCl, 2.7 mM KCl, 1.2 mM NaHCO_3_, 0.36 mM NaH_2_PO_4_, 1 mM CaCl_2_, 5 mM HEPES, 5.5 mM glucose), activated for 10 min with 2.5 µg/mL collagen (in stirring condition), 500 ng/mL convulxin (static) or saline (static), and further incubated for 30 min at RT with either Alexa Fluor 488 anti-C3aR1 (clone hC3aRZ1, AbD Serotec), fluorescein isothiocyanate (FITC) anti-CD88 (clone P12/1, AbD Serotec) or a FITC control IgG. The expression of C3aR1 and C5aR was measured by flow cytometry.

### Statistical analyses

All experiments were performed in at least three independent series of experiments. A one-way ANOVA (unequal variances) and pairwise comparisons (LSD Method) were used to compare changes between the different groups. Results are presented as means and standard error of mean (SEM). Analyses were performed with SPSS 14.0 (SPSS Inc. Chicago, IL, USA) and PRISM-5 softwares (GraphPad Software, USA). A p-value of 0.05 was considered significant.

## Results

### C5b-9 formation during platelet aggregation

**Figure 1:**
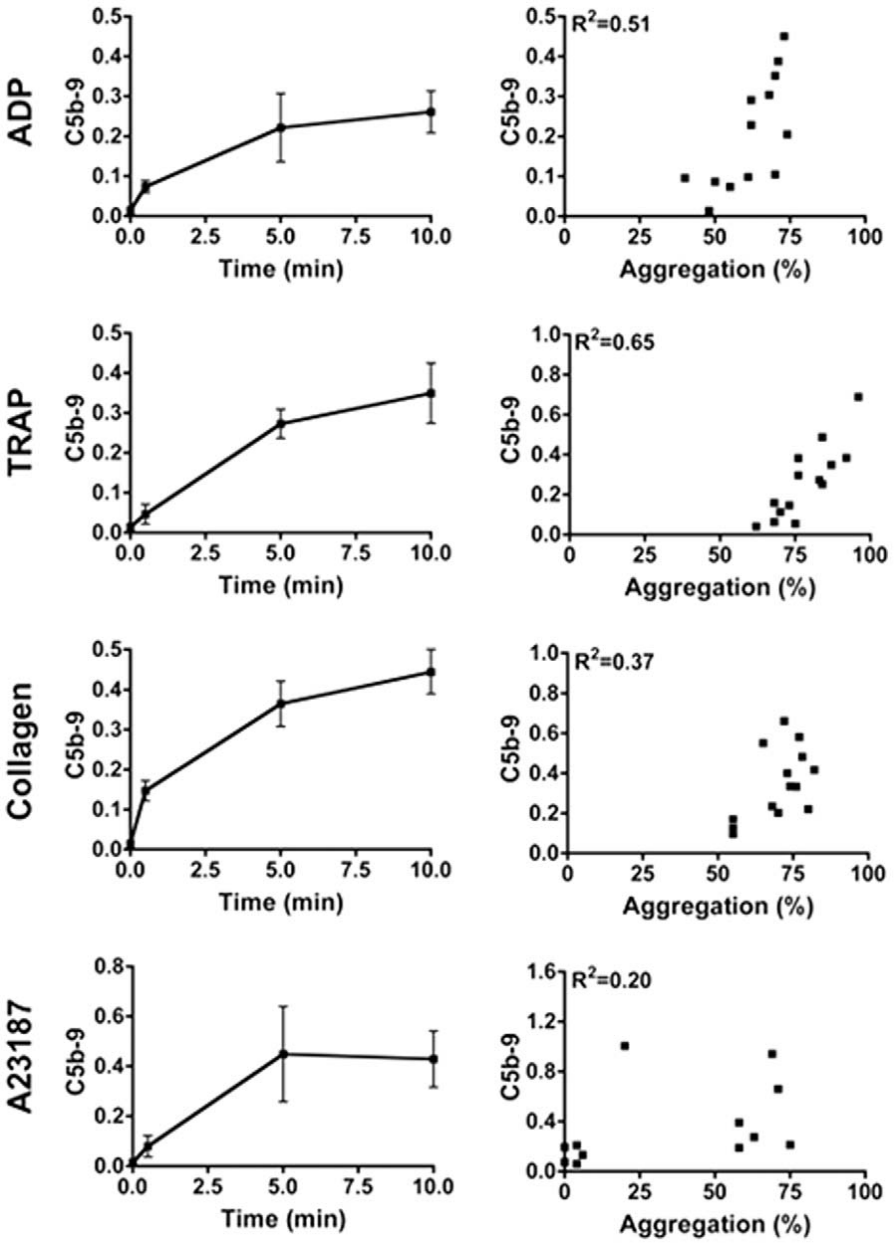
Kinetics of C5b-9 formation on activated platelets. Platelets and aggregates from PRP stirred during 30 s, 5 min or 10 min at 37°C in the presence of ADP (5 µM), TRAP (10 µM), collagen (2.5 µg/mL) or A23189 (50 µM) were analyzed for C5b-9 formation by flow cytometry. Results are expressed as (MFI_C5b-9_/mean_FSC_) ± SEM (n = 10). The correlation between C5b-9 formation and the percentage of platelet aggregation measured at 10 min for each agonist are shown on the right. Coefficients of determination R^2^ are indicated.

Undetectable on resting platelets, C5b-9 was detected as early as 30 s following platelet activation by all agonists tested (Figure 1). C5b-9 levels reached a plateau at 5 min with collagen (0.47±0.26 MFI_C5b-9_/mean_FSC_ versus 0.52±0.17 at 10 min) and ADP (0.24±0.14 MFI_C5b-9_/mean_FSC_ versus 0.25±0.12 at 10 min), whereas there was a continuous time-dependent increase following TRAP (0.43±0.16 MFI_C5b-9_/mean_FSC_ at 10 min) and A23187 activation (0.71±0.51 MFI_C5b-9_/mean_FSC_ at 10 min).

The correlation between C5b-9 formation and platelet aggregation was greatest with TRAP (R^2^  = 0.65 versus  = 0.51 and 0.37 for ADP and collagen, respectively), whereas it was noticeably weak with A23187 (R^2^  = 0.20), which induced a heterogeneous aggregatory response (Figure 1).

### C5b-9 and P-selectin positive platelets

**Figure 2:**
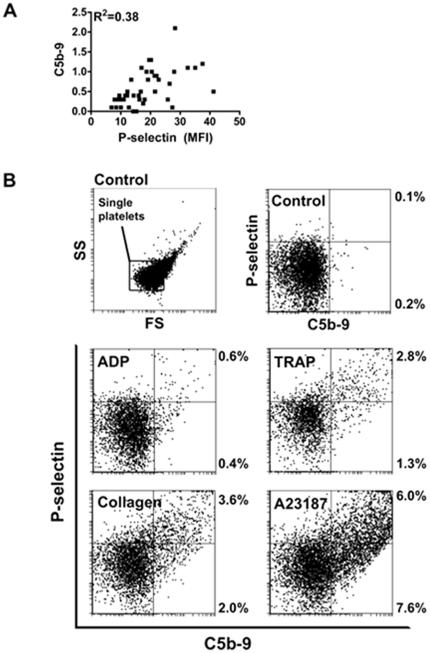
C5b-9 forms on single activated platelets independently of P-selectin. Flow cytometry analyses for C5b-9 deposition and P-selectin expression on platelets activated with ADP (5 µM), TRAP (10 µM), collagen (2.5 µg/mL) or A23189 (50 µM). A: correlation between C5b-9 (MFI_C5b-9_/mean_FSC_) values (those reported in Figure 1) and the corresponding values for P-selectin expression for each agonist. B: C5b-9 and P-selectin signals on single platelets. Analyses were restricted to a region corresponding to single platelets in a forward scatter/side scatter dot plot representation (top left panel). For each agonist, representative distributions of platelets double positive for C5b-9 and P-selectin (upper right quadrants) or positive for C5b-9 but negative for P-selectin (lower right quadrants) are shown. All values are expressed in logarithmic scales. Experiments were performed in triplicates.

Because P-selectin was shown to activate the complement system [9], we measured the co-labelling of C5b-9 and P-selectin during platelet activation. A relatively good correlation between C5b-9 formation and P-selectin expression was observed during platelet aggregation (R^2^  = 0.32) (Figure 2). However, a closer inspection of activated, but non-aggregated platelets, revealed that one third (with TRAP) to more than half (with A23187) of C5b-9-positive platelets were negative for P-selectin. After 10 min of activation, the percentage of platelets positive for C5b-9 remained low (from ∼1% with ADP to <15% with A23187).

### C5b-9 signal identifies a distinct platelet subpopulation

**Figure 3:**
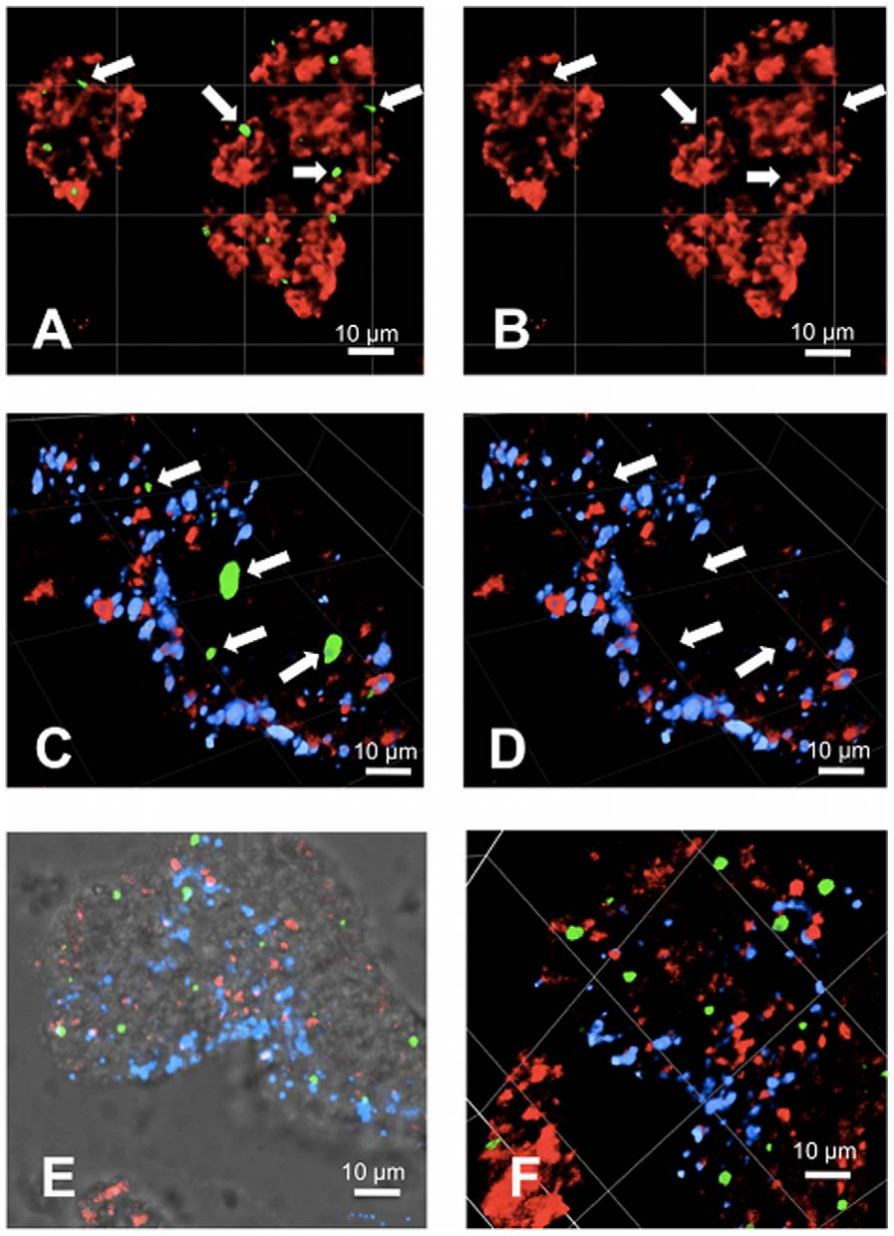
C5b-9 formation in ADP-, TRAP- and A23187-induced platelet aggregates. Confocal microscopy of ADP- (5 µM), TRAP- (10 µM) or A23189- (50 µM) induced platelet aggregates labelled for C5b-9, P-selectin and phosphatidylserine. A: tri-dimensional volume view of P-selectin (red) and C5b-9 (green) in TRAP-induced aggregates. B: same view as A with C5b-9 signal omitted. C: tri-dimensional volume view of P-selectin (red), C5b-9 (green) and annexin V (blue) staining in ADP-induced aggregates. D: same view as C with C5b-9 signal omitted. E: Two-dimensional projection view of platelets (bright field), P-selectin (red), C5b-9 (green) and annexin V (blue) in A23187-induced aggregates. F: tri-dimensional volume view of the same aggregates. White arrows point to the position of single platelets incorporated into the aggregates and exhibiting a strong C5b-9 signal.

**Figure 4:**
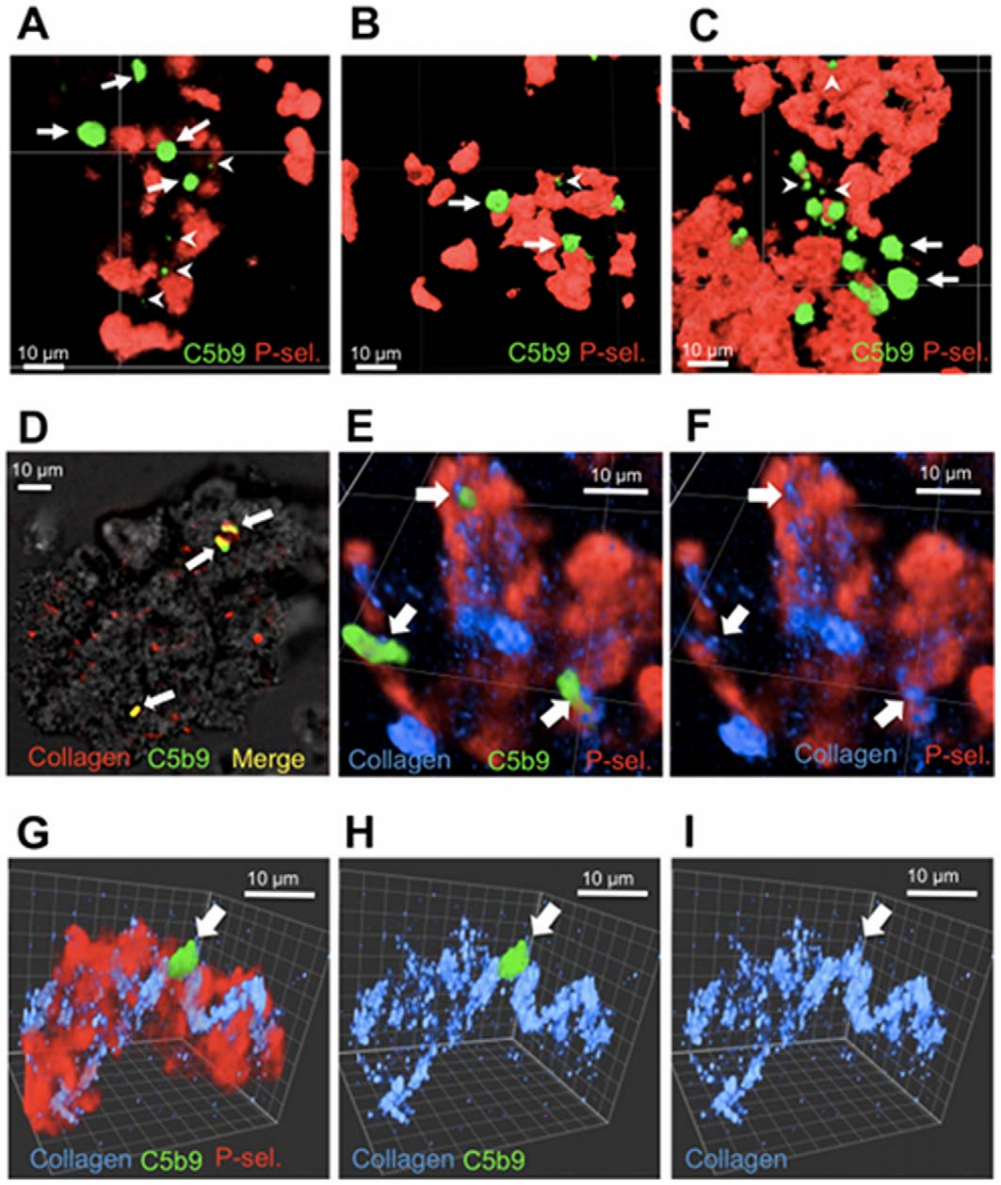
C5b-9 formation in collagen-induced platelet aggregates. Confocal microscopy of collagen-induced platelet aggregates labelled for C5b-9 deposition and P-selectin expression. Collagen fibres were visible by autofluorescence. A: two-dimensional projection view of platelets (bright field), collagen (red) and C5b-9 (green). B: example of a tri-dimensional volume view of P-selectin (red), C5b-9 (green) and collagen (blue) in an aggregate. C: same view as B with C5b-9 signal omitted. D: other example with same labels. E: same view as D with P-selectin signal omitted. F: same view as E with C5b-9 signal omitted. White arrows point to the position of single platelets exhibiting a strong C5b-9 signal.

Confocal microscopy analyses revealed that, irrespective of the agonist used, C5b-9 signal originated from only a small number of single platelets incorporated into the thrombi (Figure 3 and 4). Double labelling of C5b-9 and P-selectin on TRAP-induced aggregates showed that C5b-9-positive platelets formed a population distinct from P-selectin-positive platelets (Figure 4 A–B). Triple labelling of C5b-9, P-selectin and phosphatidylserine on ADP- and A23187-induced aggregates confirmed these observations and further revealed that C5b-9-positive platelets incorporated into aggregates were largely negative for phosphatidylserine (Figure 4 C–F). C5b-9-positive platelets were distributed randomly within ADP-, TRAP- and A23187-induced aggregates in contrast to annexin V-positive platelets which were localized on the edge regions of the thrombi (Figure 4 C–F).

### C5b-9-positive platelets associate with collagen fibres

**Figure 5:**
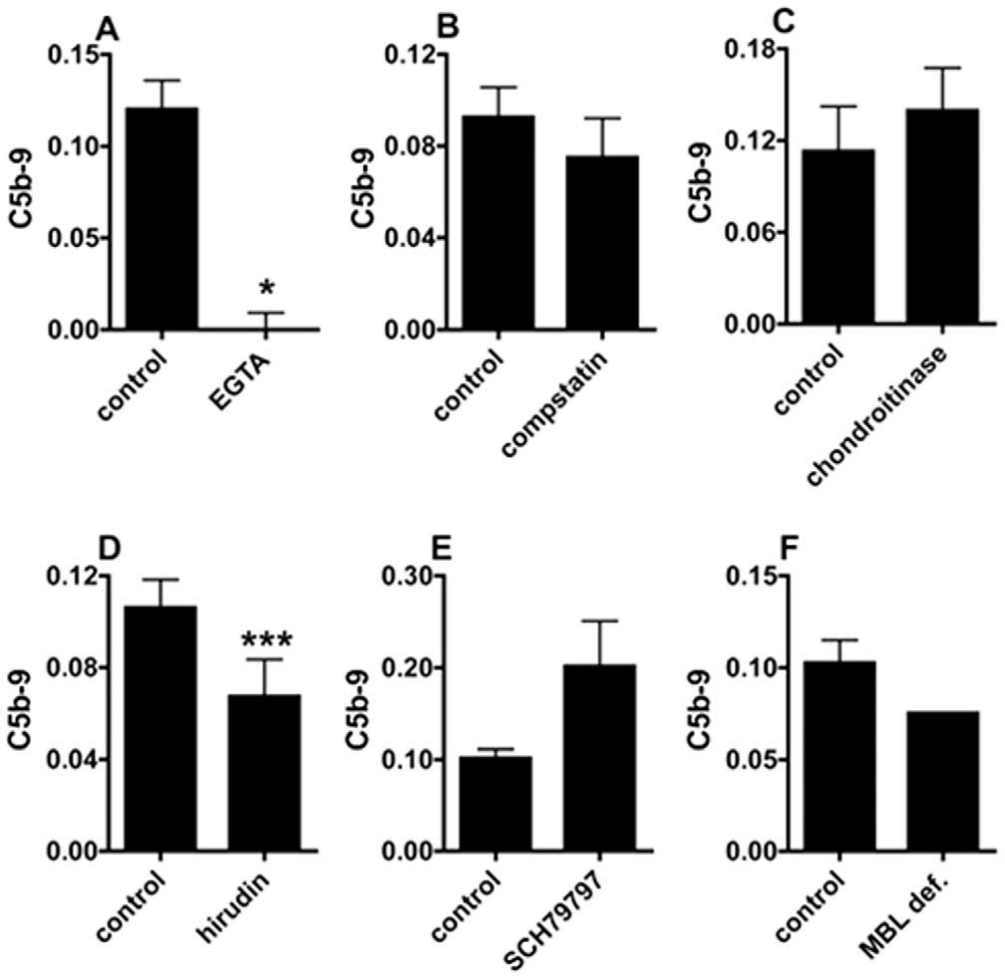
Inhibition of C5b-9 formation on collagen-activated platelets. Platelet C5b-9 formation following collagen activation in the presence of A: 10 mM Mg^2+^-EGTA (n = 4); B: 100 µM compstatin (n = 4), 5 U; C: chondroitinase (n = 6), 11 U/mL; D: hirudin (n = 16) or E: 3 µM SCH79797 (n = 7) measured by flow cytometry. In F, the C5b-9 values obtained with the PRP from a MBL deficient donor (duplicate) were compared with normal PRPs (n = 15). Results are expressed as (MFI_C5b-9_/mean_FSC_) ± SEM. *: p<0.02; ***: p<0.006.

Similarly to ADP, TRAP and A23187, C5b-9 signal in collagen-induced aggregates was restricted to few platelets (Figure 5A,B,C, arrows) and not co-localized with P-selectin. The confocal images also revealed the presence of dots of C5b-9 signal of approximately 1 µm or smaller that could correspond to microparticles (Figure 5A,B,C, arrow heads). The observation of collagen fibres that emitted auto-fluorescence at 450 nm when excited at 405 nm confirmed that C5b-9-positive platelets were localized in direct contact with collagen fibres (Figure 5D to I). Again, although the P-selectin signal was observed at the vicinity of collagen fibres, it was not strictly associated with the C5b-9 signal.

### Hirudin inhibits the formation of C5b-9 on collagen-activated platelets

We investigated the involvement of recently identified mechanisms of complement activation during platelet activation [9]-[11] in collagen-activated platelet. Mg^2+^-EGTA, used as an inhibitor of C1q-dependent complement activation, completely inhibited C5b-9 formation (Figure 5A). In contrast, the C3 inhibitor compstatin and the chondroitine sulfate-degrading enzyme chondroitinase did not inhibit C5b-9 formation (Figure 5B,C). Because EGTA also inhibits the coagulation cascade triggered by the anionic phospholipids that are expressed on activated platelets that leads to thrombin generation, we also investigated the effect of the direct thrombin inhibitor hirudin (Figure 5D). Hirudin blocked C5b-9 formation by 37% (p<0.006, n = 16), which is in contrast to the specific thrombin receptor PAR-1 inhibitor SCH79797 that slightly increased C5b-9 levels (+ 99% p = 0.08, n = 7) (Figure 5E). Finally, using the PRP from a MBL-deficient donor to investigate the involvement of the LP, we observed that C5b-9 formation was similar to that of normal PRP (Figure 5F).

### Soluble and platelet-bound anaphylatoxins

**Figure 6:**
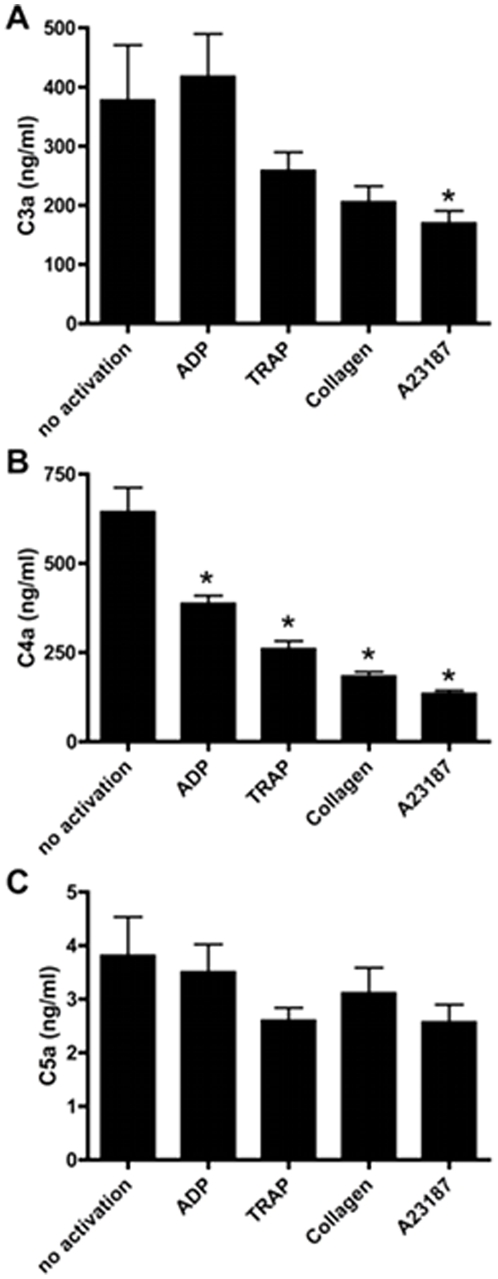
Effect of PRP activation on soluble anaphylatoxins levels. The concentrations of soluble C3a (A), C4a (B) and C5a (C) were measured in PRP activated by ADP (5 µM), TRAP (10 µM), collagen (2.5 µg/mL) or A23189 (50 µM) (n = 10). *: p<0.05.

Compared with unactivated PRP (409.9±99.1 ng/mL), soluble C3a levels remained stable following activation with ADP (442.9±75.6 ng/mL p = 0.80), TRAP (291.6±29.2 ng/mL, p = 0.28) and collagen (237.7±29.4 ng/mL, p = 0.13), but decreased significantly with A23187 (182.8±20.0 ng/mL, p<0.05) (Figure 6A). As for soluble C4a, the levels also decreased significantly following activation with ADP (392.1±25.3 ng/mL, p<0.003), TRAP (281.5±19.5 ng/mL, p<0.001), collagen (199.9±12.3 ng/mL p<0.001) and A23187 (142.8±5.9 ng/mL, p<0.001) (unactivated PRP: 681.0±69.2 ng/mL) (Figure 6B). Compared with unactivated PRP, C5a levels remained stable irrespective of the agonist used (Control: 3.9 ± 0.8 ng/mL; ADP: 3.5±0.6, p = 0.69; TRAP: 3.5±0.6, p = 0.64; collagen 3.8±0.7, p = 0.87; A23187: 2.6±0.4, p = 0.16) (Figure 6C).

**Figure 7:**
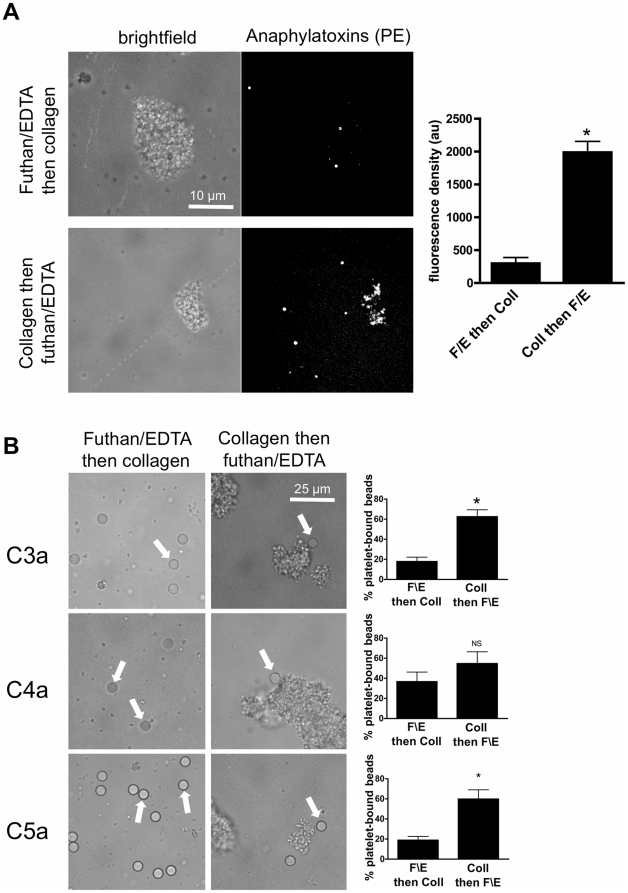
Anaphylatoxins bind to collagen-activated platelets. A. Platelet aggregates formed in PRP activated with 2.5 µg/mL collagen in the absence or presence of Futhan (50 µg/mL) and EDTA (10 mM) were washed and incubated with a PE-conjugated anti-anaphylatoxin C3a, C4a and C5a antibody cocktail (see Methods) and analysed by confocal microscopy. Representative bright field and fluorescence images of each condition are shown on the left side. The mean fluorescence densities ± SEM of at least 5 platelet aggregates measured for each condition in 3 independent assays are show on the right side. *: p<0.0001 B. Collagen-induced platelet aggregates formed in the absence or presence of Futhan and EDTA were washed and incubated with either anti-C3a, anti-C4a or anti-C5a antibody-coupled beads (see Methods). Representative bright field images of each condition are shown on the left side. Arrows are pointing to beads interacting with platelets. The expression of each anaphylatoxin on platelets was evaluated by measuring the percentage of beads bound to single platelets or aggregates in each condition. At least 5 images taken at random for each condition in at least 4 independent assays were analyzed. *: p<0.02.

To determine whether the decrease of soluble anaphylatoxins following platelet activation was due to their capture by platelets, collagen-activated platelets were harvested, washed in PBS-EDTA, incubated with the cocktail of PE-conjugated antibodies against anaphylatoxin C3a, C4a and C5a from the CBA assay (see Methods), and analyzed by confocal microscopy (Figure 7A). When platelets were preincubated with futhan and EDTA to block complement activation prior to collagen activation, only few PE signal were measured in platelet aggregates. In contrast, the activation of platelets by collagen prior to the addition of futhan and EDTA induced a strong fluorescent signal on aggregates. No fluorescence was detected on resting platelets (data not shown). To identify which anaphylatoxin(s) were bound to platelets, collagen-activated platelets were washed in PBS-EDTA, incubated with beads bearing antibodies against C3a, C4a or C5a (from the CBA assay described in Methods), and the percentage of bound beads was measured by contrast phase microscopy (Figure 7B). When futhan and EDTA were added prior to collagen activation, the binding of anti-C3a beads increased from 17.9% to 62.6% in the reversed condition (p<0.012). Similarly, the binding of anti-C5a beads to platelets increased from 18.9% to 59.8% (p<0.013). In contrast, the percentage of platelet–bound anti-C4a beads was already high when futhan and EDTA were added prior to collagen activation (36.7%), and only increased moderately in the reversed conditions (54.7%, p = 0.44).

### C3a and C5a receptors are expressed on collagen- and convulxin-activated platelets

**Figure 8:**
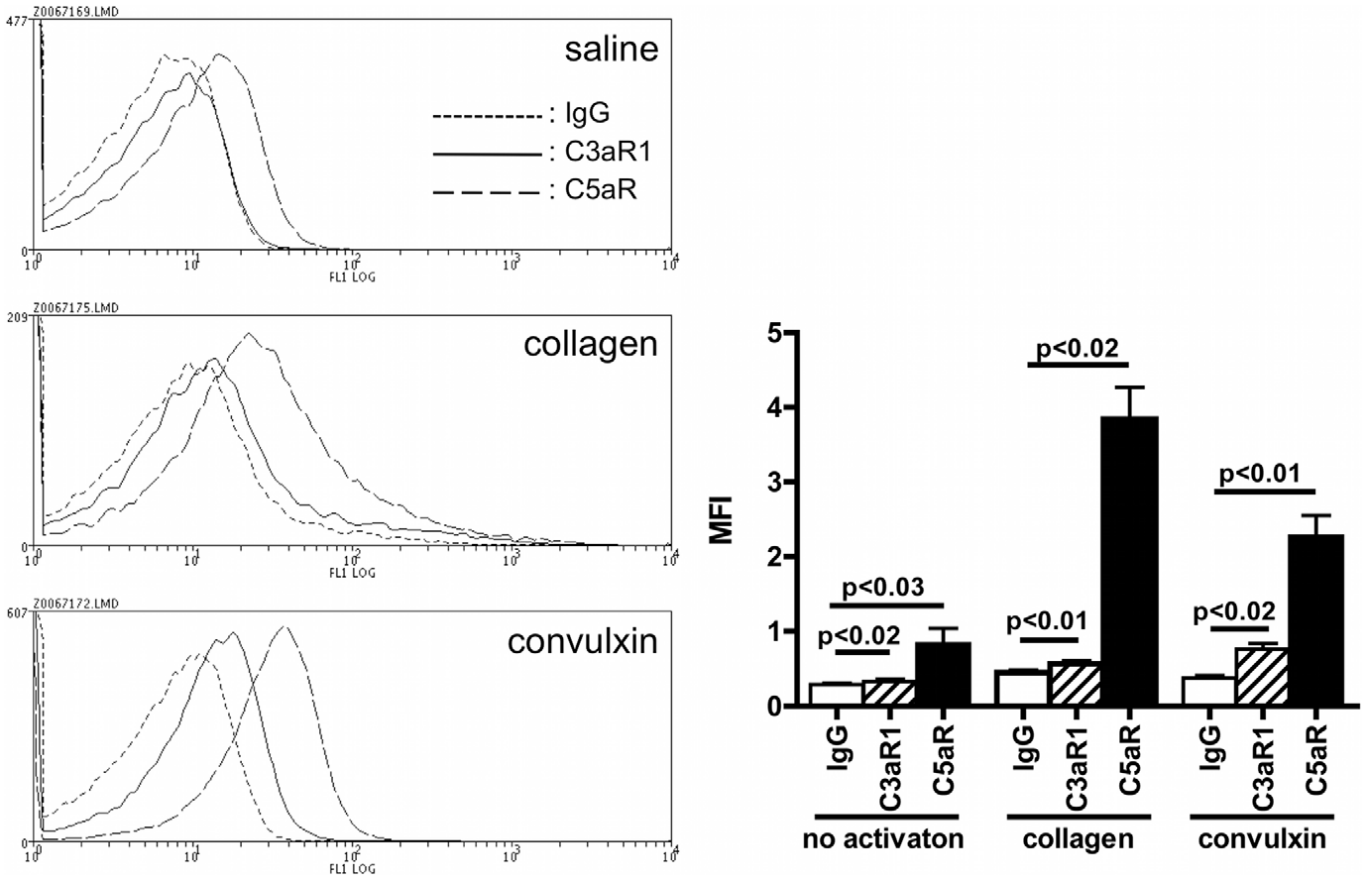
Expression of C3aR1 and C5aR on collagen- and convulxin-activated platelets. Washed platelets were activated for 10 min with 2.5 µg/mL collagen, 500 ng/mL convulxin or saline and further incubated for 30 min at RT and analyzed for C3aR1 and C5aR expression by flow cytometry. A: representative overlays of fluorescence histograms obtained for each condition using an alexa fluor 488 anti-C3aR1 (solid line), a FITC anti-CD88 (dashed line) or a FITC control IgG (dotted line). B: Mean fluorescence intensities ± SEM of 3 to 9 experiments for each condition.

The expression of the anaphylatoxin receptors C3aR1 and C5aR (CD88) were measured by flow cytometry with fluorescently-labelled monospecific antibodies on resting, collagen- (2.5 µg/ml) or convulxin- (500 ng/ml) activated washed platelets (Figure 8). C3aR1 was barely detectable on resting platelets (+14% vs control IgG, MFI, p<0.02), but its expression was up-regulated following collagen (+26%, p<0.006) or convulxin (+101%, p<0.02) activation. C5aR was readily detected on resting platelets (+185%, p<0.002), and its expression was also upregulated following collagen (+755%, p<0.002) and convulxin (+503%, p<0.005) activation.

## Discussion

The choice of the blood anticoagulant is crucial with combined experimental investigations of platelets and complement, as it may interfere with complement activation. Sodium citrate is a Ca^2+^ chelator and, similar to EDTA, may affect the C1 complex and the activation of the CP. Heparin inhibits the CP independently of its antithrombin III cofactor activity by potentiating the C1 esterase inhibitor [14] and the activation of the AP by impairing the C3 convertase activity [15]. Hence, thrombin inhibitors have been usually considered more appropriate for these investigations [16], although a recent study has demonstrated that thrombin participates in complement activation through the direct cleavage of C5 [17]. Rationalizing the choice of the anticoagulant for our study using a complement activity assay [12], we found that sodium citrate had the lowest impact on the three complement pathways, compared with EDTA, heparin and PPACK (see Supporting Information S1) and was therefore used throughout our study.

Our results indicate that platelet activation with collagen, ADP, TRAP, or A23187 induced the rapid and sustained formation of membrane C5b-9. In agreement with previous studies, this formation was characterized by strong donor inter-variability [18]. The kinetics of C5b-9 formation identified differences between agonists. With ADP, C5b-9 reached a plateau at values inferior to those observed with the other agonists at 10 min, suggesting that the cessation of C5b-9 formation was due to a regulatory process rather than a consumption of complement components.

P-selectin has been involved in C3b binding to activated platelets, CS activation and C5b-9 formation [9]. Consistently, we found that both platelet aggregation and overall P-selectin signal increased concomitantly with C5b-9 formation. However, our analyses nuanced these observations by showing that, irrespective of the agonist used, a substantial proportion of C5b-9-positive platelets were negative for both P-selectin and annexin V. Our results indicate that P-selectin-dependent activation of the AP is not solely responsible for C5b-9 formation on platelets. Platelets express several binding sites for the CP components, notably C1q which binds cC1qR on the platelet membrane and triggers CS activation. However, C1q also induces α_IIb_β_3_ activation, P-selectin and phosphatidylserine exposure [19] and, therefore, is unlikely directly responsible for the formation of C5b-9 on platelets negative for P-selectin and phosphatidylserine.

Irrespective of the agonist used, we observed a relatively low number of C5b-9-positive platelets incorporated into the thrombi. These platelets were mostly found in direct contact with collagen fibres within collagen-induced aggregates, arguing for a direct contribution of collagen on CS activation. These platelets exhibited a compact and spherical shape with apparent maintenance of integrity, which is in contrast to annexin V-positive platelets that had an inflated and deformed shape. These observations argue against a direct link between phosphatydilserine exposure and C5b-9 assembly on the same platelet.

In an attempt to understand the process of C5b-9 formation that accompanies collagen-induced platelet aggregation, we used inhibitors targeting recently proposed platelet-related mechanisms of complement activation [9]–[11], [17]. Several arguments point towards the direct involvement of thrombin in this process. First, we unexpectedly observed that the potent thrombin inhibitor PPACK obliterated the activation of the AP in plasma. Second, the specific thrombin inhibitor hirudin, inhibited C5b-9 formation on collagen-activated platelets, which is in contrast to the C3 inhibitor compstatin, chondroitinase or MBL deficiency. The lack of effect of compstatin, a direct inhibitor of C3, implies that thrombin action takes place downstream of C3 in the complement activation cascade. In addition, the lack of an inhibitory effect of SCH79797, a potent inhibitor of thrombin-mediated PAR-1 activation, on C5b-9 formation argues against a PAR-1-mediated action of thrombin in this process. Overall, these observations echo the study of Huber-Lang and collaborators demonstrating that thrombin may substitute for the C3-dependent C5 convertase in C3-deficient mice by directly cleaving C5 in plasma [17]. The authors demonstrated that C5a was reduced by antithrombin III or hirudin in bronchoalveolar fluids of inflamed lungs, but only when C3 was absent thus suggesting that complement activation by thrombin may constitute a compensatory adaptive pathway. Relative to these findings, our observation (using the Wielisa compl300 kit) that PPACK abolished the AP-dependent C5b-9 formation may have been exacerbated by the presence of Mg^2+^EGTA in the assay which was used concomitantly to LPS to block the CP [12].

Hence, the results of our study show that, both *in vitro* and in the presence of sodium citrate, thrombin contributes to C5b-9 formation on collagen-activated platelets even when all three conventional complement activation pathways are functional. In light of these findings, the obliteration of C5b-9 formation on platelets that we observed using Mg^2+^-EGTA could be interpreted as the inhibition of C1q-dependent CP activation, but also as the inhibition of thrombin generation that requires Ca^2+^ and anionic phospholipid surfaces to occur. Our finding that C5b-9 formation was not strictly associated with phosphatidylserine exposure further suggests that thrombin formed on procoagulant platelets may activate the complement in the fluid phase, thus leading to the formation of C5b-9 on distal platelets. The recent observation by Hamad and collaborators that, under physiological conditions, complement activation occurred in the vicinity of TRAP-activated platelets, but not on their surface, supports this hypothesis [20].

Platelet activation by soluble C5b-9 was previously shown to generate large amounts of C5b-9-bearing microparticles, suggesting that microparticles were selectively shed from the plasma membrane at the site of C5b-9 insertion [21]. In addition, platelet microparticles in contact with plasma express C5b-9 on their surface [22]. In our study, and in agreement with previous studies involving the complement terminal pathway in platelet microparticle formation [21], collagen induced a two-fold increase of microparticle count; increase that was inhibited by compstatin and Mg^2+^-EGTA (supporting information S2, Figure S2A,B). Interestingly, chondroitinase had no effect (Figure S2B), suggesting that chondroitine sulphate-triggered complement activation in the fluid phase [11] is not instrumental to collagen-induced platelet microparticle formation.

Compared with unstimulated PRP, we observed a two-fold increase of C5b-9 content in microparticles isolated from collagen-activated PRP, but no significant increase with the other platelet agonists (Figure S2C,D). It is therefore unlikely that, in our experimental conditions, the extent of C5b-9 shedding was sufficient to explain the presence of only a small subset of platelets per aggregates bearing strong C5b-9 signal. On the other hand, it is expected that a substantial fraction of the microparticles formed during platelet activation in stirred condition for ten minutes incorporates into platelet aggregates [23]. The confocal images of collagen-induced aggregates revealed a C5b-9 signal originating from the structures of sub-micron diameter that may indeed correspond to microparticles.

Platelet subpopulations have been reported by Dale and collaborators who described the production of highly procoagulant platelets following the co-stimulation with thrombin and collagen (coated platelets) [24]. Kulkarny and Jackson described the formation of a related platelet subset forming on collagen surfaces under flow condition (SCIP, sustained calcium-induced platelet morphology) with procoagulant and pro-inflammatory properties [25]. Because both coated platelets and SCIP are characterized, among other markers, by phosphatidylserine exposure, it is likely that the C5b-9-positive platelets identified in the present study constitute a distinct platelet subset. Further investigations are necessary to understand the mechanism of their formation and their exact role in the intricate interplay between complement and haemostasis.

Complement activation leads to the formation of anaphylatoxins C3a, C4a and C5a. Unexpectedly, we could not detect any increase of soluble anaphylatoxin levels during platelet activation. Rather, we observed a decrease of soluble C3a with A23187 and of soluble C4a with all agonists whereas C5a levels remained stable with all agonists.

In order to overcome the intervariability of separate assays, we have chosen to harvest and freeze the supernatants of activated PRP from each donor and to perform a grouped measurement of anaphylatoxin levels (n = 10 donors) on the same day and on the same plate. For this purpose, we have used a well standardized and sensitive commercial assay (Cytometric Bead Array immunoassay, BDTM). To ascertain that the slow thawing of samples would not interfere with the measurement, we also performed a side experiment in which anaphilatoxins levels were measured in PRP rapidly thawed at 37°C (4 donors, unstimulated versus collagen-stimulated conditions). In these conditions, we observed similar tendencies as those presented in the present manuscript (personnal data). It is therefore unlikely that the decrease of anaphylatoxin levels following platelet activation was due to inappropriate sample handling.

The present results are slightly different from those of del Conde and collaborators who also reported no significant increase of C3a with ADP and TRAP, but an increase following collagen activation of citrated PRP [9]. They are also different from those of Hamad and collaborators who reported an increase of C3a in hirudinized PRP activated with TRAP [11]. The method of detection, the shorter duration of activation used by the first group (1 min) and the different anticoagulant and agonist used by the second group might explain these differences.

We hypothesized that the decrease of soluble anaphylatoxins was due to their binding to activated platelets. To date, very little is known regarding the binding of anaphylatoxins to human platelets. C3a and C5a were not shown to interact with resting human platelets [26], [27]. Our data confirm that only few, if any, anaphylatoxins bind to resting platelets. Importantly, our study is, to our knowledge, the first to report the binding of C3a, C4a and C5a to thrombi formed following platelet activation. Two C3a receptors - C3aR1 and C3aR2 - and two C5a receptors - C5aR (CD88) and C5L2 - have been identified on other cell types [28], [29], but no information on the putative C4a receptor on human cells is available. C5aR have been detected in platelet lysates by western blot, but not on the surface of resting platelets by flow cytometry [30]. Our results show that C5aR, and to a lesser extent C3aR1, are expressed on platelets activated by collagen or convulxin and could, thereby, contribute to the binding of C5a and C3a to platelets.

In conclusion, we suggest that the exposure of collagen fibres and the generation of thrombin at sites of vessel wall injury will directly participate in the local activation of the complement by platelets. It is also expected that the anaphylatoxins generated locally will accumulate in the platelet thrombus. Such interactions could be of physiologic importance for platelet function, but also for the control and localization of inflammation at sites of thrombosis.

## Supporting Information

Figure S1 **Effect of anticoagulation on complement pathways activation in plasma.** Using a solid phase enzyme immunoassay of C5b-9 formation, the functionality of the CP, AP and LP was measured in the plasma of healthy donors with no MBL deficiency anticoagulated with either sodium citrate, EDTA, PPACK or heparin. Results are expressed as the mean % of positive control (n = 4, duplicates).
(TIF)

Figure S2 **Platelet microparticle formation and C5b-9 content.** A: Microparticles formed in the PRP activated by ADP (5 µM), TRAP (10 µM), collagen (2.5 µg/mL) or A23189 (50 µM) as measured by flow cytometry (n = 3 to 5). B: Inhibitory effect of 10 mM Mg^2+^-EGTA, 100 µM compstatin, 5 U chondroitinase, 11 U/mL hirudin or 3 µM SCH79797 on collagen-induced microparticles formation. C: C5b-9 content in microparticles following platelet activation detected by western blot in reduced conditions using antibody AE11 recognizing a neoepitope on C9 (≈66 kDa). Results are expressed as the mean ± SEM of integrated density (n = 5). A representative western blot pattern is shown. * p<0.05. D: the formation of microparticles (10 donors) following PRP activation with ADP (5 µM), TRAP (10 µM), collagen (2.5 µg/mL) or A23189 (50 µM) were pooled and analysed as in C in non-reduced condition for the detection of poly-C9. The western blot pattern is shown.
(TIF)

Supporting Information S1 (DOC)
