# Supplementary figures and images for: Requirements for Membrane Attack Complex Formation and Anaphylatoxins Binding to Collagen-Activated Platelets
Source: PLoS One. 2011 Apr 15;6(4):e18812. doi: 10.1371/journal.pone.0018812 (PMC3078139; doi:10.1371/journal.pone.0018812)

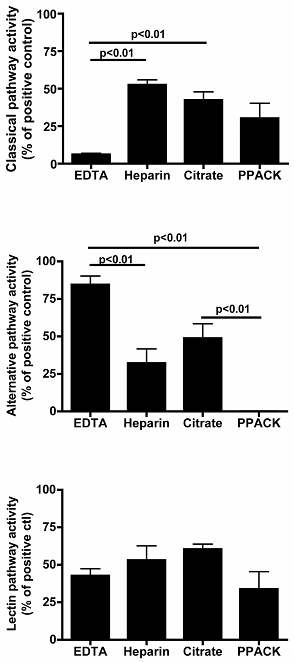

Supplement: Figure S1 — Effect of anticoagulation on complement pathways activation in plasma. Using a solid phase enzyme immunoassay of C5b-9 formation, the functionality of the CP, AP and LP was measured in the plasma of healthy donors with no MBL deficiency anticoagulated with either sodium citrate, EDTA, PPACK or heparin. Results are expressed as the mean % of positive control (n = 4, duplicates). (TIF) [file pone.0018812.s001.tif]

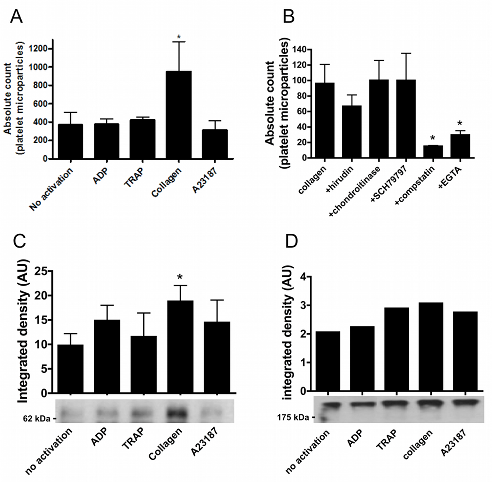

Supplement: Figure S2 — Platelet microparticle formation and C5b-9 content. A: Microparticles formed in the PRP activated by ADP (5 µM), TRAP (10 µM), collagen (2.5 µg/mL) or A23189 (50 µM) as measured by flow cytometry (n = 3 to 5). B: Inhibitory effect of 10 mM Mg2+-EGTA, 100 µM compstatin, 5 U chondroitinase, 11 U/mL hirudin or 3 µM SCH79797 on collagen-induced microparticles formation. C: C5b-9 content in microparticles following platelet activation detected by western blot in reduced conditions using antibody AE11 recognizing a neoepitope on C9 (≈66 kDa). Results are expressed as the mean ± SEM of integrated density (n = 5). A representative western blot pattern is shown. * p<0.05. D: the formation of microparticles (10 donors) following PRP activation with ADP (5 µM), TRAP (10 µM), collagen (2.5 µg/mL) or A23189 (50 µM) were pooled and analysed as in C in non-reduced condition for the detection of poly-C9. The western blot pattern is shown. (TIF) [file pone.0018812.s002.tif]
