# Supplementary material for: Requirements for Membrane Attack Complex Formation and Anaphylatoxins Binding to Collagen-Activated Platelets
Source: PLoS One. 2011 Apr 15;6(4):e18812. doi: 10.1371/journal.pone.0018812 (PMC3078139; doi:10.1371/journal.pone.0018812)
Supplement: Supporting Information S1 — (DOC) [file pone.0018812.s003.doc]

**Supporting information S1**

***Determination of the anticoagulant for blood sampling***

Because the blood anticoagulant may substantially interfere with complement activation, we compared, as a preliminary step of our study, the effect of the classical anticoagulants sodium citrate, EDTA, heparin and PPACK on the plasma activation of the CP, AP and LP.

Blood samples from healthy volunteers with no MBL deficiency (serum MBL values > 100 ng/mL as measured by ELISA) and free from any medication known to interfere with platelet function and complement activation was drawn from the antecubital vein in plastic tubes containing either sodium citrate (0.105 M), ethylenediaminetetraacetic acid (EDTA), D-phenylalanyl-L-prolyl-L-arginine chloromethyl ketone (PPACK) or heparin.

After discarding the first 2 mL, the remaining 18 mL were centrifuged at 1800g for 15 min at room temperature (RT), and the functionality of the classical (CP), lectin (LP) and alternative (AP) complement pathways was assessed using a solid phase enzyme immunoassay for C5b-9 formation (Wielisa kit COMPL 300 Wieslab, Lund, Sweeden) [12]. Briefly, diluted plasmas were incubated at 37°C in microtiter wells precoated with IgM, LPS or mannan for the activation of the CP, AP and LP, respectively. After washing, the wells were incubated for 30 min at RT with an alkaline phosphatase-conjugated anti-human C5b-9 neoantigen. After a further washing step and incubation with a p-nitrophenyl phosphate substrate, the absorbance was measured at 405 nm. Expressed as a % of positive control, the median reference values given by the manufacturer for sera samples were 100%, 73% and 56% for the CP, AP and LP, respectively.

Our results (Figure S1) confirmed that EDTA strongly interfered with the CP (5.5% of residual activity), while heparin and sodium citrate were associated with the lowest negative impact on CP (49.8% and 47.7%, respectively versus 28.5% for PPACK). The AP activation was minimally affected by EDTA (80.5%) and only to some extent by sodium citrate (51.5%). It was, however, strongly reduced by heparin (23.0%) and obliterated by PPACK (0.03%). Compared with the median normal reference value for sera (56%), the LP was only moderately affected by either anticoagulant,, with values ranging from 28.2% for PPACK to 62.2 % for sodium citrate. On the basis of these results, sodium citrate was selected as the blood anticoagulant for the study.

***C5b-9 expression on platelet microparticles***

Microparticles generated during PRP activation by a variety of platelet agonists (diameter between 0.5 and 0.9 µm) were quantified by flow cytometry in the harvested PPP using the Megamix kit (Biocytex, Marseille, France).

Platelet microparticles were harvested by ultracentrifuging the activated PPPs twice at 100,000g for 1 h at RT in a calcium/magnesium-free Tyrode’s–Hepes buffer. Microparticle-protein concentrations were measured using the BCA assay (Fischer Scientific, Ottawa, ON, Canada), and 20 μg per sample were run on a 10 % sodium dodecyl sulfate-polyacrylamide gel with or without 100 µmol/L dithiothreitol. Western blotting analyses were performed using the anti-human C5b-9 antibody AE11 (1:100 dilution) and a secondary peroxidase-conjugated goat anti-mouse IgG (Bio-Rad Laboratories, Mississauga, ON, Canada). The Chemiluminescence Reagent Plus (Perkin Elmer, Waltham, MA, USA) was used for detection. Washed resting platelets were used as a negative control for C5b-9 formation.

We compared the amount of microparticles formed during the 10-minute activation of PRP with each agonist, and analysed the C5b-9 content. Collagen induced a two-fold increase in the microparticle count, whereas ADP, TRAP and A23187 resulted in an effect similar to that observed in unstimulated PRP (Figure S2A). Compstatin and Mg^2+^-EGTA inhibited collagen-induced microparticle formation by 84% (p<0.02) and 69% (p<0.04), respectively, whereas hirudin was associated with a non-significant reduction (-30%, p=0.09). Chondroitinase and SCH79797 did not show any inhibitory activity (Figure S2B). Compared with unstimulated PRP, and greater than all other agonists, the analysis of C5b-9 content showed an approximately two-fold increase in C9 neoepitope (reduced condition) in microparticles produced by collagen (Figure S2C). The detection of high molecular weight poly-C9 in non-reduced conditions confirmed the greater amount of C5b-9 present in collagen-induced microparticles (Figure S2D). Noticeably, C5b-9 was detected in the microparticles from non activated PRP.
